# Altered Heart Rate Variability in Spontaneously Hypertensive Rats Is Associated with Specific Particulate Matter Components in Detroit, Michigan

**DOI:** 10.1289/ehp.1002831

**Published:** 2010-12-15

**Authors:** Annette C. Rohr, Ali Kamal, Masako Morishita, Bhramar Mukherjee, Gerald J. Keeler, Jack R. Harkema, James G. Wagner

**Affiliations:** 1 Electric Power Research Institute, Palo Alto, California, USA; 2 University of Michigan, Ann Arbor, Michigan, USA; 3 Michigan State University, East Lansing, Michigan, USA

**Keywords:** air pollution, cardiac function, heart rate variability, particulate matter, toxicology

## Abstract

**Background:**

Exposure to fine particulate matter [aerodynamic diameter ≤ 2.5 μm (PM_2.5_)] is linked to adverse cardiopulmonary health effects; however, the responsible constituents are not well defined.

**Objective:**

We used a rat model to investigate linkages between cardiac effects of concentrated ambient particle (CAP) constituents and source factors using a unique, highly time-resolved data set.

**Methods:**

Spontaneously hypertensive rats inhaled Detroit Michigan, CAPs during summer or winter (2005–2006) for 13 consecutive days. Electrocardiogram data were recorded continuously, and heart rate (HR) and heart rate variability (HRV) metrics were derived. Extensive CAP characterization, including use of a Semicontinuous Elements in Aerosol Sampler (SEAS), was performed, and positive matrix factorization was applied to investigate source factors.

**Results:**

Mean CAP exposure concentrations were 518 μg/m^3^ and 357 μg/m^3^ in the summer and winter, respectively. Significant reductions in the standard deviation of the normal-to-normal intervals (SDNN) in the summer were strongly associated with cement/lime, iron/steel, and gasoline/diesel factors, whereas associations with the sludge factor and components were less consistent. In winter, increases in HR were associated with a refinery factor and its components. CAP-associated HR decreases in winter were linked to sludge incineration, cement/lime, and coal/secondary sulfate factors and most of their associated components. Specific relationships for increased root mean square of the standard deviation of successive normal-to-normal intervals (RMSSD) in winter were difficult to determine because of lack of consistency between factors and associated constituents.

**Conclusions:**

Our results indicate that specific modulation of cardiac function in Detroit was most strongly linked to local industrial sources. Findings also highlight the need to consider both factor analytical results and component-specific results when interpreting findings.

Numerous epidemiological and toxicological studies have reported associations between ambient fine particulate matter [aerodynamic diameter ≤ 2.5 μm (PM_2.5_)] and adverse cardiovascular events (e.g., Chen et al. 2010; Lanki et al. 2006). A number of these studies have documented changes in heart rate variability (HRV) (e.g., Schwartz et al. 2006). However, PM_2.5_ is a complex mixture of both organic and inorganic materials, and the specific components responsible for these changes are unclear. The use of ambient particle concentrators, coupled with repeated measures and extensive exposure characterization, has facilitated identification of causative PM components. Investigators have reported linkages with water-soluble metals and organic compounds (Kodavanti et al. 2005), silicon (Si) and organic carbon (OC) (Batalha et al. 2002), vanadium (V) and bromine (Saldiva et al. 2002), and other trace elements (Gurgueira et al. 2002). Other investigators have employed factor analytical and other source apportionment methodologies to try to identify the sources of PM influencing toxicological outcomes (e.g., Chen et al. 2010); however, to date, these methods have been more widely employed in epidemiological studies (e.g., Sarnat et al. 2008).

The development of semicontinuous monitoring methodologies for elemental analysis has facilitated the identification of PM components linked with health effects. In particular, these methods allow the collection of highly time-resolved exposure measurements to correspond to continuous cardiac function data in animals. The Semicontinuous Elements in Aerosol Sampler (SEAS) (Kidwell and Ondov 2001, 2004) uses high-resolution inductively coupled plasma–mass spectrometry (ICP-MS) to perform every-30-min multielemental analysis of PM_2.5_ samples. The increased temporal resolution of the data—and thus the number of data points—coupled with repeated measures on individual animals, increases the statistical power to observe an effect. The research reported here represents the first time this instrument has been used in the context of continuous physiological data.

Our group has conducted a number of air quality and health studies at Maybury Elementary School in southwest Detroit, Michigan (e.g., Harkema et al. 2009; Keeler et al. 2005). This area is characterized by heavy motor vehicle traffic, particularly diesel trucks, because of its proximity to the Ambassador Bridge and interstate freeways. In addition, there is dense industrial activity upwind of the site, including iron/steel manufacturing, coke ovens, chemical plants, refineries, sewage sludge incineration, and coal-fired utilities. Morishita et al. (2006) found that allergic airway responses to concentrated ambient particles (CAPs) were likely associated with local combustion sources, including refineries and incinerators, but were independent of sulfate and total mass. More recently, we reported that the mild pulmonary and systemic changes observed in CAP-exposed rats were likely due to local PM_2.5_ sources such as oil combustion, oil refining, waste incineration, and traffic (Rohr et al. 2010).

In the present study, we sought to determine the associations between specific PM components and source factors and altered cardiac function in CAP-exposed rats. We conducted an exposure similar to that reported previously (Rohr et al. 2010), but we used twice the number of animals per group. In addition, we utilized a SEAS sampler and used those data to estimate source factors. We then determined associations between PM component data, as well as source factors, for HRV parameters.

## Materials and Methods

### Site description

This study was conducted during 16–28 July 2005 and 11–23 February 2006 at Maybury Elementary School in southwest Detroit, Michigan. Multiple exposure assessment and toxicological studies have been conducted at this location (e.g., Harkema at al. 2009; Keeler et al. 2005; Morishita et al. 2006).

### Mobile laboratory/concentrator

We used AirCARE1, a 53-ft mobile laboratory outfitted with extensive air quality monitoring equipment and inhalation exposure chambers, constructed collaboratively by Michigan State University and the University of Michigan. The concentrator performance and the exposure systems in AirCARE1 have been described previously (Harkema et al. 2009; Sioutas et al. 1997).

### Exposure characterization

Integrated CAP mass was determined during each 8-hr exposure period using 47-mm Teflon (polytetrafluoroethylene) filters (Gelman Science, Ann Arbor, MI) in Teflon/Teflon-coated aluminum filter packs (URG Corp., Chapel Hill, NC) affixed to the back of the animal exposure chamber. Sodium carbonate–coated backup filters were placed behind the Teflon filters to correct for volatilization losses of nitrate. Prebaked quartz filters (Gelman Science) were placed in URG filter packs mounted on the exposure chamber and sampled at flow rates of 2 L/min. CAP mass was also measured continuously (every 5 min) using a TEOM (tapered element oscillating microbalance) 1400a ambient particulate monitor (Rupprecht and Pataschnick Inc., Albany, NY). Black carbon was monitored using an aethalometer (Magee Scientific, Berkeley CA).

We monitored ozone using a continuous ultraviolet photometric analyzer (Teco 49; Teco Diagnostics, Anaheim, CA), carbon monoxide (CO) using a nondispersive infrared analyzer (Teco 48S), nitrogen oxides using a commercial chemiluminescence detector (Teco 42S), and sulfur dioxide (SO_2_) using a pulsed fluorescence technique (Teco 43S).

Meteorological parameter data (temperature, relative humidity, precipitation, wind speed, wind direction, and solar radiation) were collected to evaluate the temporal variability in local transport pathways and source influences.

We used annular denuder/filter pack systems to determine concentrations of major ions in CAPs, including sulfate, nitrate, ammonium, and acidity. Elemental carbon (EC) and OC were determined by a thermal–optical analyzer (Sunset Laboratories, Tigard, OR) using NIOSH Method 5040 [National Institute for Occupational Safety and Health (NIOSH) 2003]. For trace element analysis, PM samples collected on Teflon filters were wetted with ethanol and extracted in 10% nitric acid solution. The extraction solution was sonicated for 48 hr in an ultrasonic bath and then allowed to passively acid-digest for a minimum of 2 weeks. The details of the SEAS have been previously described in detail (Kidwell and Ondov 2001, 2004); slurry samples from SEAS were also acidified in 10% nitric acid and stored in the refrigerator for a minimum of 2 weeks. Sample extracts were then analyzed for a suite of trace elements using ICP-MS (ELEMENT2; Thermo Finnigan, San Jose, CA). All aerosol sampling was performed using clean techniques.

### Positive matrix factorization analysis

We used U.S. Environmental Protection Agency (EPA) Positive Matrix Factorization (PMF) 3.0 (U.S. EPA 2010) to investigate source factors; PMF is a variant of factor analysis that constrains factor loadings and factor scores to nonnegative values and has been described in detail (Paatero and Tapper 1994). The summer and winter data sets consisted of 268 and 236 SEAS 30-min samples, respectively. The results of the summer field campaign were described in detail by Morishita et al. (2011). The elemental concentration and analytical uncertainties for each element for each sample, together with the sampling uncertainties, were input into the model. The optimal solution was determined by multiple model runs to examine the effect on the numbers of factors assigned and the different FPEAK (rotational parameter) values on the range of results that were both physically reasonable and where the objective function *Q*-value does not change substantially. The FPEAK value was set at zero, where the value of robust *Q* reaches a global minimum.

### Animal exposures

Sixteen male spontaneously hypertensive rats (Charles River Laboratories, Portage, MI) 13–14 weeks of age, were used in the study. Animals were treated humanely and with regard for alleviation of suffering, and were maintained and used in accordance with National Institutes of Health guidelines. All protocols were approved by the All University Committee on Animal Use and Care at Michigan State University. Rats were initially housed in animal facilities at Michigan State University until moved to the mobile laboratory, where they were housed two per polycarbonate cage on corncob bedding with *ad libitum* access to food and water. Exposures were carried out in two stainless-steel Hinners-type whole-body inhalation chambers; one received CAPs, and the other received HEPA-filtered clean air at the same flow rate as the experimental group. Eight rats were exposed in each chamber from 0700 hours to 1500 hours for 13 consecutive days. Light cycles were set on a 12/12-hr light/dark cycle beginning at 0600 hours. Temperature and relative humidity in the chambers were monitored and are reported in Table 1 for both air- and CAP-exposed rats. In the summer, mean temperature was 24.9°C for air-exposed rats and 22.5°C for CAP-exposed rats (*p* < 0.0001 by *t*-test). In the winter, mean temperatures were 25.5°C and 24.5°C for air- and CAP-exposed rats, respectively (*p* = 0.012). There were no significant differences in relative humidity between air- and CAP-exposed rats (85.1% vs. 83.3% in summer for air and CAP exposures, respectively; 41.0% vs. 35.9% in winter).

### Electrocardiogram (ECG) monitoring and telemetry

Two to 3 weeks before exposures, rats were surgically implanted with bipotential PhysioTel transmitters (TA11CTA-F40; Data Sciences International, St. Paul, MN) that emit radio signals of ECGs, heart rate (HR), and temperature. Transmitters were placed intraperitoneally with leads terminating in a lead II configuration. Telemetry receivers were modified and affixed inside individual cages in exposure chambers that were customized for telemetry studies. Data streams of 30-sec duration were collected every 5 min during exposures.

### ECG analysis

Automated ECG analysis was applied to the recorded signals using Physiostat ECG Analysis data analysis software (Data Sciences International) to generate the raw data for HRV analysis. We used the R-R intervals for all normal beats (N-N intervals) to calculate time-domain measures of HRV: standard deviation of the N-N intervals (SDNN) and square root of the mean squared differences of successive N-N intervals (rMSSD).

### Statistical analysis

To evaluate differences in HR and HRV measures between CAP-exposed and control rats (*n* = 8/group), we used mixed models in SAS (version 9.1; SAS Institute Inc., Cary, NC); this approach accounts for the longitudinal nature of the measurements on each animal. For those HRV measures that were determined to be significantly different between exposure groups (*p* < 0.05), univariate regression analyses were carried out to determine which CAP components and/or gaseous copollutants were associated with changes in cardiac function.

## Results

### Composition of exposure atmospheres

The exposure composition for the summer sampling campaign was described in detail, along with receptor modeling results, by Morishita et al. (2011).

#### Summer (July 2005)

Table 1 shows CAP mass and major component concentrations for each summer exposure day. Mean CAP concentration for the summer exposure period was 518 μg/m^3^ (range, 68–1,638 μg/m^3^). PM composition showed high variability, with organic matter (OM) concentrations ranging from 6 to 381 μg/m^3^ and sulfate ranging from 6 to 648 μg/m^3^. Mean nitrate and ammonium concentrations were 27 μg/m^3^ and 57 μg/m^3^, respectively. “Unidentified mass,” mass not accounted for by the component categories in Table 1, would include some portion of OM and particle-bound water. In the summer, this fraction was 23% of PM mass, whereas in the winter it was 15%. Based on the composition of the PM, most of the unidentified mass was likely particle-bound water because relative humidity during the exposures was typically high (mean = 62%). Detailed data on elemental composition are provided in Supplemental Material, Table 1 (doi:10.1289/ehp.1002831).

During the summer exposure period, high ambient PM_2.5_ concentrations were associated primarily with southwesterly winds [see Supplemental Material, Figure 1a (doi:10.1289/ehp.1002831)], illustrating the strong impact of local emission sources located on and around the Zug Island heavy industrial complex southwest of the study site.

*R*^2^ values for correlations between major PM components and overall mass were high for ammonium (0.97) and sulfate (0.92), moderate for nitrate (0.67) and OM (0.68), and low for EC (0.04). Average ambient gas concentrations were relatively low. Mean CO, SO_2_, and nitric oxide (NO) concentrations were 0.5 ± 0.2 ppm, 5.2 ± 4.1 ppb, and 5.9 ± 12.8 ppb, respectively.

#### Winter (February 2006)

In winter, CAP mass averaged 357 μg/m^3^, which was lower than in summer (Table 1). Both OM (56 μg/m^3^) and sulfate (47 μg/m^3^) were also lower in winter, but, as expected, nitrate (98 μg/m^3^) and ammonium (65 μg/m^3^) concentrations were higher. Winter elemental concentrations [see Supplemental Material, Table 1 (doi:10.1289/ehp.1002831)]were generally lower than in summer, with the exception of a few earth [magnesium (Mg), aluminum (Al), potassium (K)] and trace [V, molybdenum (Mo)] elements. Average CO, SO_2_, and NO concentrations were 0.3 ± 0.2 ppm, 6.0 ± 4.5 ppb, and 16.5 ± 18.8 ppb, respectively. In the winter, high ambient PM_2.5_ concentrations were primarily associated with southwesterly and northeasterly winds [see Supplemental Material, Figure 1b). However, mean ambient PM_2.5_ for the entire exposure period was only about 9 μg/m^3^, and we observed less temporal variability than during the summer.

The correlation between PM mass and sulfate was weaker in winter than in summer (*R*^2^ = 0.60), whereas the correlation between mass and EC was stronger (*R*^2^ = 0.22). Mass and nitrate were highly correlated (0.89), reflecting the significant contribution of nitrate to PM mass in Detroit in this season.

### Source apportionment analyses

We identified source factors using PMF methods as described by Morishita et al. (2011) for the summer season. Combined with wind directionality and correlation analyses with gaseous pollutants and EC, we identified six factors: secondary aerosol, gasoline- and diesel-powered vehicles, sludge incineration, refining, cement/lime production, and iron/steel manufacturing. For the contribution of each element to each of the identified factors, see Supplemental Material, Figure 2a (doi:10.1289/ehp.1002831).

In winter, we applied the same methods to the data set and resolved six factors: coal/secondary sulfate, gasoline- and diesel-powered vehicles and iron/steel manufacturing, sludge incineration, refining, cement/lime production, and metal processing and iron/steel manufacturing. For the contribution of each element to these factors, see Supplemental Material, Figure 2b (doi:10.1289/ehp.1002831). Because of the lack of semicontinuous OC data, we could not evaluate the OC contribution to the secondary sulfate factor; however, other research suggests that this factor likely also contains secondary organic aerosol(Blanchard et al. 2008). Because of low ambient PM_2.5_ mass concentrations and less temporal variability of elemental concentrations in winter, we observed more mixtures of source factors than in summer.

We observed some similarities in factor composition between seasons (e.g., gasoline- and diesel-powered vehicles, refineries, and cement/lime production) but also some differences [e.g., sludge incineration, iron/steel manufacturing; see Supplemental Material, Figure 2 (doi:10.1289/ehp.1002831)]. Sewage sludge in Detroit is a by-product of treatment of both impervious-surface runoff and some industrial discharge to the sewers. Thus, variability in industrial inputs may have contributed to the observed seasonal differences in the sludge incineration factor. Variability in the iron/steel factor may be at least in part due to the change of ownership of one of the iron/steel manufacturing industries near the site (formerly the Rouge Steel Company) in 2004; the facility did not resume full operation until 2006.

### ECG/cardiac function

Mean values for HR [beats per minute (bpm)], SDNN, and rMSSD in summer and winter in air- and CAP-exposed rats are presented in Table 2.

#### Summer: 8-hr data

Mixed models were used to determine if there were significant differences between CAP-exposed and control rats for any of the ECG parameters. In the summer, we found no significant differences in HR, SDNN, or rMSSD (data not shown). Given the lack of exposure-related effects, we did not determine associations with CAP constituents.

#### Summer: 30-min data

**Figure 1:**
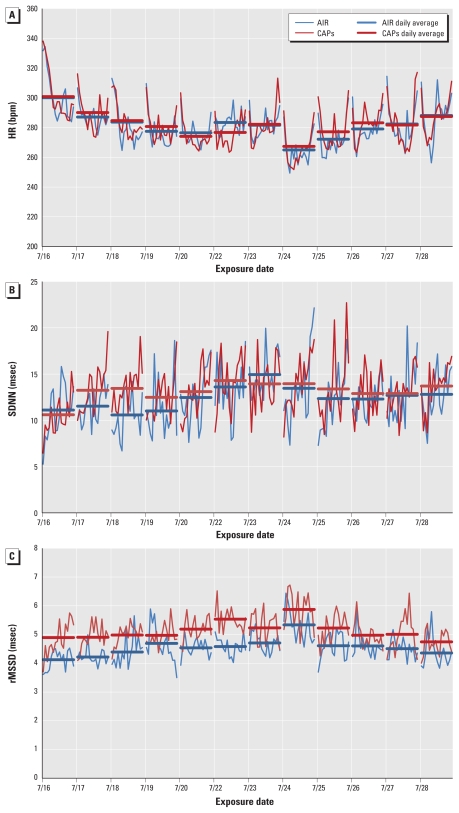
Temporal variability in HR (*A*), SDNN (*B*), and rMSSD (*C*) for air- and CAP-exposed rats, July 2005. Thick lines indicate daily means; thin lines indicate 30-min means for all rats.

Using the more highly time-resolved data set, *p*-values for HR, SDNN, and rMSSD differences between air and CAP-exposed rats were 0.06, 0.003, and 0.85, respectively. Figure 1 shows the temporal pattern of changes in HR, SDNN, and rMSSD over the 13-day summertime exposure period, providing both daily means and 30-min data to illustrate the significant variability, both within a given day and across days.

**Figure 2:**
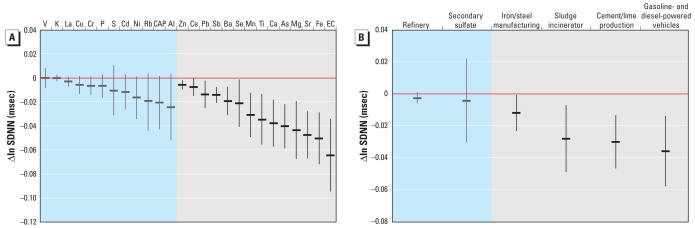
Associations between SDNN and CAP constituents (*A*) and PMF source factors (*B*) in summer, expressed as change per interquartile range of the constituent or factor. Effect estimates in gray sections are statistically significant (*p* < 0.05).

Reductions in SDNN were associated with EC, iron (Fe), strontium (Sr), Mg, arsenic (As), calcium (Ca), titanium (Ti), manganese (Mn), selenium (Se), barium (Ba), antimony (Sb), lead (Pb), cesium (Ce), and zinc (Zn) (Figure 2A). We observed significant associations between reduced SDNN and iron/steel manufacturing, sludge incineration, cement/lime production, and gasoline- and diesel-powered vehicle factors (Figure 2B).

Given the significant temperature differences between air- and CAP-exposed rats, we ran additional models controlling for chamber temperature. Although temperature was an independent predictor of HR and HRV, it was independent of the group effect (air vs. CAPs) and did not change the pollutant-specific effect estimates.

#### Winter: 8-hr data

In the winter, only HR showed a significant exposure-related effect (*p* = 0.0194) using the 8-hr integrated data. With this data set, increased HR was associated with Mg, whereas reduced HR was associated with Fe, Ti, copper (Cu), Pb, tin (Sn), cobalt (Co), EC, OC, Se, and indium (In) (data not shown).

#### Winter: 30-min data

Similar to our summer findings using highly time-resolved data, we found significance in mixed models not evident in the 8-hr data set. Both HR and rMSSD were significantly different between exposed and control animals (*p* < 0.0001 and *p* = 0.0278, respectively). SDNN was not significantly different between groups (*p* = 0.2478).

**Figure 3:**
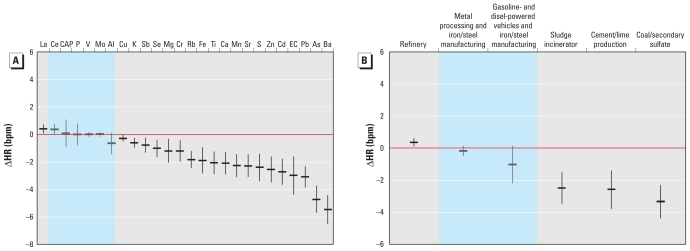
Associations between HR and CAP constituents (*A*) and PMF source factors (*B*) in winter, expressed as change per interquartile range of the constituent or factor. Effect estimates in gray sections are statistically significant (*p* < 0.05).

Increased HR (Figure 3A) was associated with lanthanum (La), whereas reductions in HR were associated with Ba, As, Pb, EC, cadmium (Cd), Zn, sulfur (S), Sr, Mn, Ca, Ti, Fe, rubidium (Rb), chromium (Cr), Mg, Se, Sb, K, and Cu. We observed significant associations between increased HR and the refinery factor, and between decreased HR and sludge incineration, cement/lime production, and coal/secondary sulfate factors (Figure 3B).

**Figure 4:**
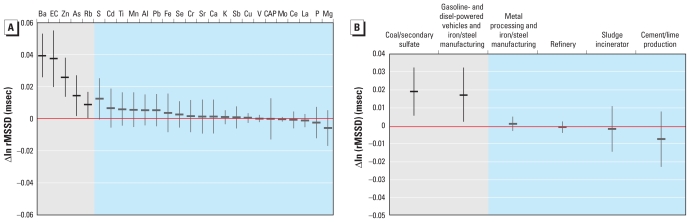
Associations between rMSSD and CAP constituents (*A*) and PMF source factors (*B*) in winter, expressed as change per interquartile range of the constituent or factor. Effect estimates in gray sections are statistically significant (*p* < 0.05).

Increased rMSSD was associated with Ba, EC, Zn, As, and Rb (Figure 4A) and with coal/secondary sulfate and gasoline- and diesel-powered vehicles and iron/steel manufacturing factors (Figure 4B).

As in the summer season, controlling for chamber temperature did not change the estimates for group effect (air vs. CAPs) or the pollutant-specific effect estimates.

## Discussion

In a similar but limited study at the same location using half as many subjects (Rohr et al. 2010), we attempted to make comparisons with CAP composition using 8-hr integrated samples. Although we detected exposure-related differences in the lung, we failed to find significant changes in cardiac function. In the present study with a larger sample size, we collected semicontinuous (every 30 min) elemental data to directly compare with HRV data in the same discrete time frame. At the same time, we also collected conventional 8-hr samples. Using this improved approach of high temporal resolution for the first time with either humans or animals, we found novel and divergent effects of CAP exposure on cardiac rhythm that depended on season and PM composition. These effects reached significance more often for the 30-min data set. Our study illustrates the utility and advantages of using more highly time-resolved exposure and outcome data, which not only increases statistical power in mixed models because of an increased sample size but also captures the often significant variability in pollutant concentrations over the course of the day.

Applying PMF to the elemental data, we linked specific source factors with acute cardiac responses in CAP-exposed rats. Specifically, we were able to link factors of motor vehicles and local emission sources unique to southwest Detroit to altered HR and HRV. These findings agree with our recent studies using less robust exposure metrics, which found that local emissions sources upwind of the exposure site, including incinerators, refineries, and metal processing operations, were linked to cardiopulmonary effects, including pulmonary deposition of metals (Morishita et al. 2006; Rohr et al. 2010). With the exception of one association we found for a coal/secondary sulfate factor in the winter, the present study also shows that similar local sources, including cement/lime production, sludge incineration, refineries, iron/steel, and motor vehicles influence HRV metrics. When the predominant wind direction is from the southwest, as in the summer, the site is affected by mixtures from multiple sources. Among the numerous sources located in that general direction, it appears that those located on a heading of 210° to 230° from the study site (I-75 corridor, sewage sludge incinerator, iron/steel manufacturing, and cement/lime production) were most strongly associated with reduced SDNN in the summer.

One of our more striking findings is the divergent responses between seasons in the direction of HR and HRV changes associated with elements and factors, despite broadly similar source factors. Specifically, we observed increased HR (though marginally significant) and decreased HRV in relation to air pollution in the summer, consistent with PM effects reported in other animal studies as well as epidemiology (Chen and Hwang 2005; Schwartz et al. 2006). However, we observed the opposite response in the winter, with decreased HR and increased HRV. Decreases in HRV concomitant with increased HR can be interpreted as greater sympathetic influence compared with parasympathetic (vagal) input (Rowan et al. 2007). SDNN is associated with overall autonomic tone of HR and is nonspecific to sympathetic versus vagal control. Therefore, our findings of depressed SDNN with source factors in summer do not suggest a specific mechanism of HRV modulation, merely a homeostatic disruption in vagosympathetic balance. However, because rMSSD is generally associated with vagal pathways, our results in winter with increased rMSSD suggest that an increase in vagal tone led to the observed decreased HR.

It is interesting that we found opposing responses from the same source factors in Detroit, albeit in different seasons. In the summer, the motor/diesel factor was linked to decreased HRV (SDNN), whereas in the winter, the combined motor vehicle/iron and steel factor was linked to increased HRV (rMSSD) (Figures 2B, 4B). The two HRV parameters do not represent exactly the same physiological process, and the factors in the two seasons differed (the winter factor was combined with iron and steel manufacturing); nevertheless, this is consistent with previously reported seasonal variations in PM_2.5_-induced oxidative stress (Becker et al. 2005). Correlations between OC and EC in summer and winter resulted in *R*^2^ values of 0.22 and 0.67, respectively. As discussed by Robinson et al. (2003) the weak correlation in summer indicates the larger contribution of secondary organic aerosol. In general, reductions in HRV are better predictors of poorer cardiac outcomes than are increases in HRV in clinical settings; therefore, we can speculate that the secondary particulate products formed in the summer may be of particular importance from a health perspective. In addition, we observed the same opposite associations for EC by season, consistent with EC being a marker for mobile source emissions. Directional differences in associations with EC could be explained by the significantly different seasonal air pollution profile in Detroit and the differing behavior of atmospheric constituents at different temperatures. For example, at colder temperatures, semivolatiles will partition more into the condensed phase and thus will be present at higher concentrations on EC particles.

To better understand source factor–HRV relationships and determine if a particular finding was robust, we looked for source factors as well as those PM constituents most heavily loaded onto the factors to be significantly associated with the same health end points. In general, we found these associations to be fairly consistent. For example, SDNN reductions in the summer were tied to iron/steel, sludge incineration, cement/lime, and motor vehicle factors. The iron/steel factor was loaded with Fe, Pb, and Zn, all of which were significant in univariate analyses, and the motor vehicle factor was loaded with a number of elements, including Fe, Ti, Mg, Cr, Mn, and As, some of which were significantly associated with HRV metrics. Previous studies have shown some of these metals to be associated with motor vehicle emissions: Mg, Ca, Fe, Zn, Ce, Pb (tailpipe emissions); Ba, Fe, Sb, and Mn (brake wear dust); and Zn (tire wear dust) (Garg et al. 2000; Schauer et al. 2006). Furthermore, EC, widely used as a marker for diesel emissions, was also significantly associated with SDNN reductions. EC, although not included in the PMF modeling, had the highest correlation with the motor vehicle factor among the six factors (Morishita et al. 2011). However, we also observed some discrepancies in the source and component results. For example, Al was loaded onto the motor vehicle factor but did not show a significant relationship with SDNN.

In winter, comparisons of source factor and component associations with cardiac end points were somewhat internally consistent, although less so for rMSSD than for HR. Only four elements, along with EC, were associated with increased rMSSD: Ba, Zn, As, and Rb. The combined motor vehicle and iron/steel factor was loaded with Rb, Al, Cr, Mn, Zn, and Ba. Therefore, three of six elements showed internal consistency. EC was also significantly associated with increased rMSSD, strengthening our confidence in the association. Of the nine elements highly loaded on the coal/secondary sulfate factor (Rb, Cd, Sb, Pb, S, V, Zn, As, Se), which was significantly associated with rMSSD, only three were associated with rMSSD in univariate models (Rb, Zn, As). Several of these elements (e.g., S and Se) that are commonly regarded as tracers of coal combustion were not associated with rMSSD. Based on these findings, we did not observe a clear and robust association between a source factor and rMSSD, although the association was stronger for the motor vehicle and iron/steel factor and its constituents than for the coal/secondary sulfate factor.

These findings, taken together, suggest the need for caution when interpreting the results of factor analyses when applied to health data sets. Both individual constituent relationships and source factor analyses should be considered; source factor analyses in isolation may not present a complete picture. To our knowledge, only one other toxicological study (Chen et al. 2010) and one epidemiological study (Sarnat et al. 2008) have compared the two types of analyses. Chen et al. (2010) concluded that component-specific analyses provide insights beyond those attainable considering only source factors; Sarnat et al. (2008) observed generally similar findings, but their analyses were not entirely comparable to ours because of the use of tracers (we considered all elements in univariate models).

Several other toxicological studies have evaluated HRV in conjunction with PM components and/or source factors, although none with the high time resolution reported in this study. In Tuxedo, New York, Lippmann et al. (2005) found significant associations between resuspended soil, secondary sulfate, and residual oil factors and HRV parameters. Other work at the same location (Lippmann et al. 2006) suggested a significant effect of nickel (Ni) on HRV in ApoE^−/−^ mice. We did not observe an association between HR and HRV parameters and Ni, and concentrations were relatively comparable; however, the New York exposures were much longer than our study. In our summer exposures, average Ni was 53 ng/m^3^, with a peak of 211 ng/m^3^ on 1 day, whereas in the New York study the mean Ni concentration was 43 ng/m^3^, with Ni peaks of about 175 ng/m^3^ on 14 days. Those 14 days had unusually low PM mass and V, a pattern that was opposite from what we observed on the high-Ni day (high V and high PM mass). In the winter season in Detroit, Ni was very low, with > 60% of the values below our assay limit of detection.

Our large data set, consisting of 208 exposure and outcome values per animal, covers only the exposure period at present. It would be of great interest to analyze nighttime, nonexposure data as well, and we plan to conduct such a secondary analysis. Other investigators have observed significant responses during nighttime hours (Chen and Hwang 2005; Lippmann et al. 2005).

Our results suggest that multiple PM components play a role in acute cardiac responses in rats exposed to Detroit CAPs. Our findings also illustrate the need to consider, in a holistic manner, both component-based and source-factor–based analyses when interpreting data. Taken together, these two approaches can yield valuable information regarding the relative toxicities of different constituents. The work reported here describes a novel and powerful tool to link highly time-resolved exposure data with health outcomes, thus providing a significant improvement in our ability to identify the sources and biological effects of specific PM components.

## Figures and Tables

**Table 1:** CAPs major components, 16–28 July 2005 and 11–23 February 2006.

										Temp (°C)		RH (%)	
Date	CEF	Mass	OM^a^	EC	Sulfate	Nitrate	Ammonium	Urban dust^b^	Unidentified	Air	CAPs	Air	CAPs
Summer													

7/16/05	15	576	109	2.6	149	29	55	21	211	23.9	20.9	99.1	99.3
7/17/05	65	1,437	269	4.9	379	152	190	44	399	25.6	22.0	93.7	95.6
7/18/05	41	1,638	381	10.1	648	64	252	45	237	24.5	23.1	88.1	91.6
7/19/05	28	360	236	7.4	33	15	20	35	13	24.9	23.3	86.7	83.3
7/20/05	13	255	132	15.6	13	10	43	31	25	25.0	23.2	86.0	79.3
7/21/05	39	594	142	3.4	121	37	57	23	212	23.7	23.0	83.1	86.7
7/22/05	26	314	121	4.3	45	8	22	26	88	25.1	21.9	82.2	81.9
7/23/05	16	115	61	2.9	6	2	7	14	24	24.9	21.9	80.5	72.8
7/24/05	7	81	50	5.0	6	4	6	13	0.0	26.4	24.1	80.3	78.1
7/25/05	5	68	31	1.4	11	1	8	16	0.0	26.2	23.7	78.0	73.0
7/26/05	31	843	339	6.1	186	17	92	30	173	24.2	22.1	86.6	88.9
7/27/05	23	213	185	8.2	11	6	12	20	0.0	24.9	22.0	80.7	78.9
7/28/05	23	240	6	1.6	5	12	6	40	169	24.2	21.9	81.4	73.3
TWA	25	518	159	5.7	124	27	57	28	120	24.9	22.5	85.1	83.3

Winter													

2/11/06	46	564	65	4.9	94	229	145	22	4	25.9	23.8	63.1	53.3
2/12/06	41	126	39	5.6	30	16	16	18	2	26.9	26.0	50.7	41.6
2/13/06	41	262	34	2.3	51	62	7	40	36	26.2	25.2	43.4	37.6
2/14/06	29	565	57	5.7	69	215	148	42	27	25.8	25.2	43.1	35.4
2/15/06	20	234	48	8.0	19	52	24	36	47	26.8	26.0	44.6	37.3
2/16/06	29	691	98	3.3	153	224	51	25	37	24.6	23.6	48.3	43.6
2/17/06	36	58	16	4.7	9	2	6	16	5	25.1	23.8	40.6	33.6
2/18/06	26	116	7	1.0	15	8	10	24	51	23.7	22.9	39.9	29.0
2/19/06	20	202	23	3.7	28	36	22	36	54	24.7	23.9	30.3	28.8
2/20/06	22	323	45	5.8	34	81	38	30	91	25.0	24.1	30.7	29.8
2/21/06	20	395	41	5.4	33	96	53	29	137	24.8	24.0	30.9	30.7
2/22/06	21	703	174	16.7	56	170	151	48	87	25.8	24.8	32.6	33.3
2/23/06	23	400	80	6.7	22	88	43	34	127	25.8	24.7	34.4	33.3
TWA	29	357	56	5.7	47	98	65	31	54	25.5	24.5	41.0	35.9

Abbreviations: CEF, concentration enrichment factor (the ratio of CAPs mass concentration to ambient PM_2.5_ mass concentration); RH, relative humidity; Temp, temperature; TWA, time-weighted average. Concentrations are given in μg/m^3^.

a OM was estimated from OC × 1.8.

b Urban dust: (1.89 × Al) + (1.4 × Ca) + (1.43 × Fe) + (2.14 × Si), where Si is estimated by *K*/0.15.

**Table 2:** Mean ± SD HR and HRV parameters by season and exposure status.

Exposure	*n*	HR (bpm)	SDNN (msec)	rMSSD (msec)
Summer				

Air	76^a^	283.7 ± 12.6	17.3 ± 2.5	4.6 ± 0.8
CAPs	104	285.3 ± 13.1	18.5 ± 3.1	5.1 ± 1.0

Winter				

Air	53	298.1 ± 11.3	15.6 ± 2.1	4.6 ± 0.6
CAPs	102	298.0 ± 11.4	15.1 ± 2.0	4.6 ± 1.2

a *n* did not equal 104 (13 days × 8 hr) because of transmitter recording problems.
